# Adenosine regulates radiation therapy-induced anti-tumor immunity

**DOI:** 10.1186/2051-1426-3-S2-P378

**Published:** 2015-11-04

**Authors:** Erik Wennerberg, Noriko Kawashima, Sandra Demaria

**Affiliations:** 1 Department of Pathology, New York University School of Medicine, New York, NY, USA

## 

Radiation therapy (RT) induces immunogenic cell death and dose-dependent release of ATP in the tumor microenvironment (TME), triggering maturation and activation of tumor-resident dendritic cells (DCs). However, extracellular ATP is rapidly catabolized to adenosine by ectonucleotidases CD39 and CD73, which are expressed by tumor cells and immune cells in the TME. Adenosine has pleiotropic immunosuppressive effects and inhibits activation of DC and effector T cells, while promoting regulatory T cells (Tregs). Here, we tested the hypothesis that conversion of ATP to adenosine hinders generation of effective anti-tumor immunity by high dose RT, reducing its synergy with anti-CTLA-4 antibody.

BALB/c mice were inoculated s.c. with 1 x 10^5^ TSA carcinoma cells on day 0 and assigned to treatment with: (1) control mAb; (2) anti-CD73 (TY/23); (3) TY/23+anti-CTLA-4 (9H10); (4) RT; (5) RT+TY/23; (6) RT+9H10; (6) RT+TY/23+9H10. TY/23 (200 µg) was administered i.p. every 4 days starting on day 11. RT was given locally as single 20 Gy dose on day 12. 9H10 (200 µg) was given i.p. on days 11, 14 and 17. On day 18, some tumors were harvested for flow cytometry analysis of DC and T cells. Mice were monitored for tumor growth/regression.

In irradiated tumors, CD73-blockade reduced the percentage of Tregs within the tumor-infiltrating CD4^+^ T cell population (7.9±2.5% in RT+TY/23 vs 20±0.8% in RT, p < 0.01) while increasing CD8^+^T cells (38.3±0.1% in RT+TY/23 vs 17.3±4% in RT, p < 0.05). Among intratumoral DCs (CD11c^+^MHCII^+^), the CD8a^+^ DC subpopulation was increased after CD73-blockade (37.9±15.7% in TY/23+RT vs 11.3±4.9% in RT, p < 0.01). Importantly, in irradiated mice, TY/23-administration enhanced activation of DCs and effector T cells, shown by increased CD40 expression on CD8a^+^ DCs (MFI: 218±1 in RT+TY/23 vs 54±41 in RT, p < 0.05) and increased CD69 expression on CD8^+^ T cells (MFI: 513±126 in RT+TY/23 vs 148±59 in RT, p < 0.01). TY/23 and 9H10 given alone or in combination had no effect on tumor growth. However, each antibody potentiated tumor inhibition obtained with RT (p=0.08 for RT+TY/23 and p < 0.05 for RT+9H10 vs RT). Moreover, blockade of both CD73 and CTLA-4 in combination with RT further improved tumor control resulting in complete tumor regression in 2/5 mice (p < 0.01 for RT+TY/23+9H10 vs RT).

Our findings indicate that adenosine regulates the ability of RT to induce anti-tumor immunity, affecting both DC maturation and T cell activation. Data suggest that CD73-blockade is a promising strategy to improve synergy of RT and immunotherapy.

